# Side effect profile similarities shared between antidepressants and immune-modulators reveal potential novel targets for treating major depressive disorders

**DOI:** 10.1186/s40360-016-0090-9

**Published:** 2016-10-21

**Authors:** Yu Sun, Vaibhav A. Narayan, Gayle M. Wittenberg

**Affiliations:** Neuroscience Integrative Solutions and Informatics, Janssen Research & Development, LLC, Janssen Pharmaceutical Companies of Johnson and Johnson, Titusville, NJ USA

**Keywords:** Side effect, Major Depressive Disorder, Inflammation, Antidepressant, Immune-modulator, Anti-inflammatory drug

## Abstract

**Background:**

Side effects, or the adverse effects of drugs, contain important clinical phenotypic information that may be useful in predicting novel or unknown targets of a drug. It has been suggested that drugs with similar side-effect profiles may share common targets. The diagnostic class, Major Depressive Disorder, is increasingly viewed as being comprised of multiple depression subtypes with different biological root causes. One ‘type’ of depression generating substantial interest today focuses on patients with high levels of inflammatory burden, indicated by elevated levels of C-reactive proteins (CRP) and pro-inflammatory cytokines such as interleukin 6 (IL-6). It has been suggested that drugs targeting the immune system may have beneficial effect on this subtype of depressed patients, and several studies are underway to test this hypothesis directly. However, patients have been treated with both anti-inflammatory and antidepressant compounds for decades. It may be possible to exploit similarities in clinical readouts to better understand the antidepressant effects of immune-related drugs.

**Methods:**

Here we explore the space of approved drugs by comparing the drug side effect profiles of known antidepressants and drugs targeting the immune system, and further examine the findings by comparing the human cell line expression profiles induced by them with those induced by antidepressants.

**Results:**

We found 7 immune-modulators and 14 anti-inflammatory drugs sharing significant side effect profile similarities with antidepressants. Five of the 7 immune modulators share most similar side effect profiles with antidepressants that modulate dopamine release and/or uptake. In addition, the immunosuppressant rapamycin and the glucocorticoid alclometasone induces transcriptional changes similar to multiple antidepressants.

**Conclusions:**

These findings suggest that some antidepressants and some immune-related drugs may affect common molecular pathways. Our findings support the idea that certain medications aimed at the immune system may be helpful in relieving depressive symptoms, and suggest that it may be of value to test immune-modulators for antidepressant-like activity in future proof-of-concept studies.

## Background

Major Depressive Disorder (MDD) is a disabling psychiatric disease with high life time prevalence (16.2 % among US adults) [1]. Despite advances in the treatments of MDD, 30-40 % patients are resistant to available antidepressant medications [2]. Therefore, searching for the underlying cause of treatment-resistance and finding new treatments for depression has become a priority in psychiatric research. One intriguing hypothesis suggests that pro-inflammatory neuroactive cytokines may play a significant role in the pathogenesis of at least a subgroup of MDD patients [3]. Multiple studies have shown that some MDD patients have elevated serum levels of pro-inflammatory biomarkers, such as Interleukin 1 (IL-1), Interleukin 6 (IL-6), Tumor Necrosis Factor alpha (TNF-α), and C-reactive Protein (CRP) compared to healthy controls [4–6]. Furthermore, prolonged interferon-α treatment can induce depression, indicating a causal relationship between immune response and onset of depression [7]. Interestingly, MDD patients resistant to treatment with selective serotonin re-uptake inhibitors (SSRI) were shown to have higher IL-6 and TNF-α levels than healthy controls and euthymic patients who were formerly SSRI-resistant [8].

The crosstalk between immune response and mood changes indicated by these studies raise the possibility of treating depressive symptoms with immune-modulators or anti-inflammatory medications. A double-blind, placebo-controlled, randomized clinical study demonstrated that treatment with TNF-α antagonist infliximab reduces depressive symptoms in treatment-resistant MDD patients having baseline high-sensitivity CRP (hs-CRP) concentration greater than 5 mg/L but not in patients with lower baseline hs-CRP, suggesting the potential of using immune-modulators to treat depressive symptoms in certain subgroup of MDD patients [9]. Another immune modulator, ustekinumab, targeting IL-12/23, is reported to significantly improve symptoms of depression and anxiety of patients with moderate-to-severe psoriasis in a randomized, double-blinded, placebo-controlled trial [10]. Several clinical trials have also shown that adding nonsteroidal anti-inflammatory drugs (NSAIDs) such as cyclooxygenase (COX) inhibitors to SSRI medication helps to reduce the severity of depression, indicating that anti-inflammatory drugs could augment antidepressant effects [11, 12]. However, another large-scale study evaluating the efficacy of NSAIDs as mono-therapy for late-life depression in ~2500 elderly adults showed no significant effect in reducing depressive symptoms compared with placebo [13]. Yet a recent meta-analysis pooling results from all available randomized trials testing NSAIDs and cytokine inhibitors as antidepressants showed that the pooled effect estimate suggested anti-inflammatory treatment reduced depressive symptoms compared with placebo [14].

Overall, these results support the idea that treatment targeting the elevated levels of immune/inflammatory response in some MDD patients could relieve depressive symptoms. However, more clinical studies on additional immune therapies are needed to characterize the efficacy of different treatments. This could be both costly and time-consuming. Therefore, a systematic in silico survey of the potential of repurposing immune-related medication to treat MDD patients could help ranking these alternative therapies before testing them in animal models or clinical studies. Several such methods have been proposed, showing different levels of success. One type of method is based on the hypothesis that similar ligands are likely to bind similar proteins and makes predictions of novel drug-target interactions using compound structure similarity, protein sequences of targets and known compound-protein interactions [15–18]. However such a method will miss cases where a common biological pathway or network may be perturbed, leading to a common biological effect, despite different drugs targeted different binding pockets or even different proteins in the network (e.g. targeting IL-6 vs. IL-6 receptor). Another promising approach is to use pharmacological information such as similarities in drug induced transcriptional expression signatures [19]; while this generates interesting novel findings, it is unclear whether insights gleaned in a cell-line will translate to patients *in vivo*. For example, two drugs with similar *in vitro* transcriptional expression signatures, in the human body may be concentrating in different organs, and interacting with different cell types, ultimately resulting in different biological outcomes. A recent strategy applied has focused on drug side effect profile similarities [20, 21], based on the notion than we may not know all of the biological effects of our known compounds, but similarities in mechanism within the human environment may be revealed by similarities in side effects. This method is especially useful when drugs have limited structure similarities and may not share direct targets, which is often the case between small molecular antidepressants and large molecule immune-modulators. In this study, we conduct an *in silico* survey to identify promising candidates for repurposing immune-modulators and anti-inflammatory drugs as antidepressant by first exploring the space of side effect similarities between these drugs and then searching for additional supporting evidence by comparing transcriptional expression profiles induced by these drugs in human cell lines.

## Methods

### Drug side effect data

The side effects of 996 marketed drugs were obtained from the Side Effect Resource database (SIDER 2: http://sideeffects.embl.de/) [22]. This database extracted the side effects from public documents and drug package inserts automatically and then standardized them using the MedDRA dictionary (version 14.0). Side effects of 9 additional drugs of interest (flupirtine, dextromethorphan, phencyclidine hydrochloride, piracetam, tocilizumab, siltuximab, infliximab, golimumab, ustekinumab) were extracted manually from their labels. All except 9 side effect terms from these drugs had corresponding keywords used in SIDER2. The side effect profiles of these 9 drugs were combined together with the SIDER2 data in our analysis. The side effect profile of each drug is represented by a binary vector X = (*x*_*1*_ *, x*_*2*_ *, …x*_*K*_)^T^, where each side effect term is coded 1 or 0 to represent their presence or absence in the drug label. T is the total number of side effects, which is 4201 in our analysis. Combining all the side effect profile vectors of 1005 (996 SIDER2 + 9 drugs of interest) drug, we represent the drug by side effect relationship in a 1005 x 4201 binary matrix.

### Drug side effect profile similarity analysis

To evaluate the similarity between side effect profiles of a pair of drugs represented by two binary vectors X and X’, we calculated the weighted cosine correlation coefficient as described by Takarabe, etc. [21]:

1 $$ s\left(X,X\mathit{\hbox{'}}\right)=\frac{{\displaystyle {\sum}_{k=1}^K}{w}_k{x}_kx{\mathit{\hbox{'}}}_k}{\sqrt{{\displaystyle {\sum}_{k=1}^K}{w}_k{x}_k^2}\sqrt{{\displaystyle {\sum}_{k=1}^K}{w}_kx{\mathit{\hbox{'}}}_k^2}} $$

where *w*_*k*_ is the weight function for the *k*th side effect term defined as

$$ {w}_k= \exp \left(-{d}_k^2/{\sigma}^2{h}^2\right),\kern2em k=1,2,\dots K $$

where *d*_*k*_ is the frequency of the *k*th side effect term in the data, and *K* is the total number of side effect terms in the data, *σ* is the average frequency for all the side effect terms in the data and *h* is a parameter (set to 1 in this study). This weight function gives higher weight to the less common side effects than the more common ones, because rare side effects provide more information in terms of the specific clinical effects of the drugs.

The significance of the pair-wise similarity score is estimated by comparing the original score with scores generated by shuffling the side effect labels randomly 1000 times. To focus on drug pairs that may share substantial common on or off-target effects, only drug pairs having similarity score with random shuffling *P*-value < 0.05 and sharing at least 3 uncommon side effects (weight ≥ 0.1) were retained for further analysis. We require each drug pairs to share at least 3 uncommon side effects in order to increase the likelihood that the drugs of interest are influencing common biological pathways.

Out of the 1005 drugs studied, we identified 30 antidepressants based on the KEGG Drug database annotation (http://www.genome.jp/kegg/drug/). These included drugs approved as antidepressants, drugs used as augments with other antidepressants, as well as drugs with accumulating evidence of antidepressant activity but not approved as antidepressants yet. These 30 drugs are listed in Table 1. Drugs that had side effect profiles similar to one or more of these 30 antidepressants according to their similarity scores calculated as above were selected.

**Table 1:** Antidepressants used in the analysis

KEGG Drug ID	Name	Activity	Type
D00801	Lithium	Antidepressant	Ion
D00020	Tryptophan	Antidepressant	Amino acid
D08257	Nefazodone	Antidepressant	5-HT2A-receptor antagonist
D00559	Pramipexole hydrochloride	Antiparkinsonian with Antidepressant activity	Dopamine Agonist
D01164	Aripiprazole	Augmentation Agent to other Antidepressants	Dopamine-serotonin system stabilizer
D07591	Bupropion	Antidepressant	inhibitor of dopamine and noradrenalin reuptake
D00785	Selegiline hydrochloride	Antidepressant	monoamine oxidase B inhibitor
D02580	Isocarboxazid	Antidepressant	monoamine oxidase inhibitor
D08349	Phenelzine	Antidepressant	
D08456	Quetiapine	Augmentation Agent to other Antidepressants	Multi-acting-receptor-targeting-antipsychotics
D00563	Mirtazapine	Antidepressant	Noradrenergic and specific serotonergic antidepressant
D08626	Trazodone	Antidepressant	Serotonin antagonist and reuptake inhibitor
D05523	Pizotyline	Antidepressant	Serotonin inhibitor
D01107	Milnacipran hydrochloride	Antidepressant	Serotonin– noradrenaline reuptake inhibitor
D01179	Duloxetine hydrochloride	Antidepressant	
D07793	Desvenlafaxine	Antidepressant	
D08670	Venlafaxine	Antidepressant	
D00326	Fluoxetine	Antidepressant	Selective serotonin re-uptake inhibitor
D00824	Fluvoxamine maleate	Antidepressant	
D00825	Sertraline hydrochloride	Antidepressant	
D02260	Paroxetine hydrochloride hydrate	Antidepressant	
D07704	Citalopram	Antidepressant	
D00228	Amoxapine	Antidepressant	Tetracyclic antidepressant
D07448	Amitriptyline	Antidepressant	Tricyclic antidepressant
D07727	Clomipramine	Antidepressant	
D07791	Desipramine	Antidepressant	
D07875	Doxepin	Antidepressant	
D08070	Imipramine	Antidepressant	
D08288	Nortriptyline	Antidepressant	
D08447	Protriptyline	Antidepressant	

### Connectivity map analysis

The Connectivity Map database (https://www.broadinstitute.org/cmap/) provides a collection of reference genome-wide transcriptional expression profiles from cultured human cells treated with bioactive small molecules, and a pattern-matching tool that could help discover functional connections between drugs, genes and diseases by detecting common gene-expression changes [19]. In our analysis, we queried the Connectivity Map database for drugs whose expression signatures are similar to the immune-related drugs sharing side effect similarities with antidepressants. In CMAP, the expression profiles are organized as instances labeled with unique instance identification numbers. Each instance contains a treated and control cell pair and the list of probe sets ordered by their level of differential expression between the treatment and control pair. For drugs with expression signatures in the database, we first selected the instances with the same dosage, same cell type, and ideally same batch. Then, we generated the expression signature by selecting a set of up- and down-regulated genes with more than 1.5 fold change in drug treated cells and then used it to query the database to search for drugs with a similar expression signature. First, each instance is tested for similarity with the query signature. A similarity, or connectivity score is defined, using a rank-based enrichment method utilizing the Kolmogorov-Smirnov statistic, which measures the enrichment of up- and down-regulated genes at the top and bottom of the ranked gene lists to generate a score between -1 and +1 [19]. A high positive connectivity score with the query signature indicates a similar expression profile has been found. Second, we test whether each drug represented in the database is significantly enriched for similarity to the query signature. To do this, all instances in the database are ranked according to their connectivity scores with the query signature. The enrichment score of a particular drug, with N instances in the database, is calculated using a Kolmogorov-Smirnov statistic, based on the enrichment of its set of N instances in the ranked list of all instances. A high enrichment score indicates that the drug induces an expression profile similar to the query signature. Finally, The significance of enrichment were accessed using permutation *p*-value by comparing the enrichment score with those from 100,000 sets of N instances selected at random from the set of all instances in the result.

### Softwares

**Fig. 1:**
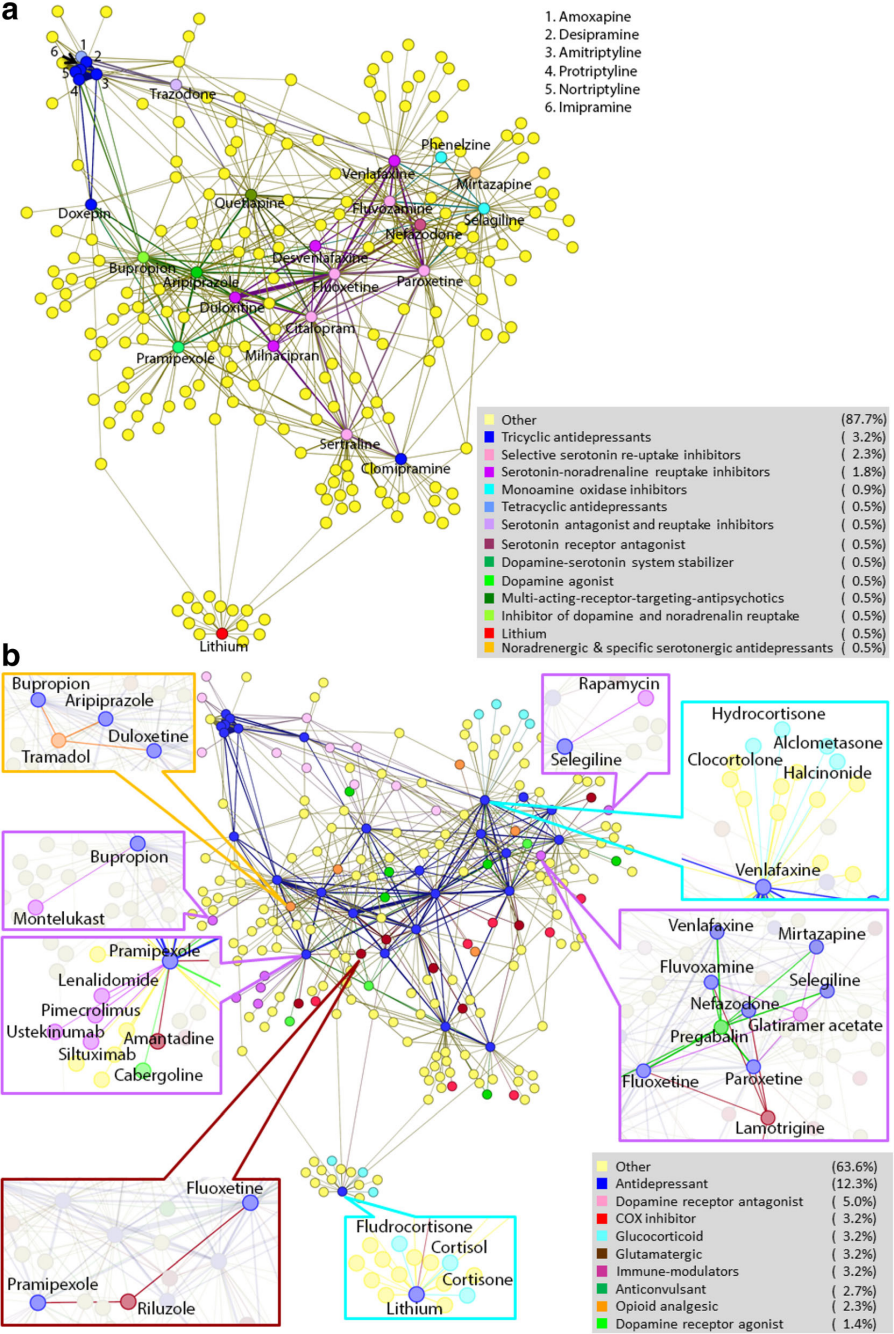
One hundred ninety drugs shared side effect profile similarities with 27 antidepressants in the study. Drugs are represented by dots and similarities between drugs are represented by edges between dots. The thickness of the edges represents the cosine correlation coefficient as determined by formula 1, which represents the degree of side effect similarity between drugs. **a** Different type of antidepressants formed different clusters according to their side effect profiles. The antidepressants are colored according to their type, while other drugs are colored yellow. **b** Anti-inflammatory drugs and immunosuppressants with side effect profile similar to antidepressants. The antidepressants are colored blue, while other drugs are colored according to their function and/or targets. Drugs that are out of the scope of this study (i.e. not immune related and have limited evidence of antidepressant-like activities) are colored yellow to simplify the background

The side effect profile similarity analysis were carried out using R 3.2.0. The similarity networks in Fig. 1 were generated using Gephi 0.8.2 beta. The connectivity map analyses were conducted using the online analysis tools at http://portals.broadinstitute.org/cmap/.

## Results

### Side effect profile similarities between antidepressants and immunological drugs

Out of the 1005 drugs studied, we identified 27 marketed antidepressants, 2 drugs (Aripiprazole and Quetiapine) used as augment to other antidepressants, and 1 drug (pramipexole) that had shown antidepressive effect but not approved yet [23] (Table 1). Using these 30 drugs as a representation of antidepressants, we identified 190 drugs having side effect profile similarities with 27 antidepressants. Three antidepressants (tryptophan, isocarboxazid and pizotyline) did not share significant side effect similarities with any of the drugs tested in our study. The similarities between these 217 drugs are depicted as a network shown in Fig. 1, in which each node represents a drug and edges between the nodes represent pair-wise side effect similarities, with the thickness of the edge corresponding to the degree of similarities. As shown in Fig. 1a, different type of antidepressants formed distinct clusters based on side effect similarities with each other. Six of the 7 tricyclic antidepressants (TCA) and 1 tetracyclic antidepressants (TeCA) formed one cluster, while 5 selective serotonin re-uptake inhibitors (SSRI) and 4 serotonin-noradrenaline reuptake inhibitors (SNRI) formed another cluster with no direct connection with the TCA cluster. Most other antidepressants were connected with the SSRI/SNRI cluster while only 4 antidepressants (trazodone, bupropion, pramipexole, and aripiprazole) shared side effect similarities with both SSRI/SNRI and TCA. Interestingly, one TCA, clomipramine, only shared side effect similarities with two SSRI but no other TCA. Lithium had a unique side effect profile and showed no significant similarities with any other antidepressants we studied.

About 48 % of the 190 drugs having side effect similarities with antidepressants have known effects on the nervous system, including dopamine receptor agonists and antagonists, GABAA-receptor agonists, μ -opioid receptor agonists and antagonists, antipsychotics, antimigraine, anticonvulsants, and analgesic drugs. Some of these drugs are already known to have antidepressant like effects. For example, tramadol, a μ-opioid receptor agonist used to treat moderate to moderately severe pain, showed side effect profile similarity with 3 antidepressants: duloxetine, aripiprazole and bupropion (Fig. 1b) . Tramadol has long been investigated for its antidepressant effect [24]. A controlled-release formulation of tramadol (ETS6103) has been developed by e-Therapeutics for treatment of MDD patients and is currently being tested in clinical trials. Two glutamate receptor signaling related drugs, riluzole (a glutamate modulator used to treat amyotrophic lateral sclerosis) and lamotrigine (a glutamate release inhibitor used for treating bipolar disorder), also showed side effect similarities with multiple antidepressants (Fig. 1b). Both have shown antidepressant-like effects in animal models and/or open-label studies [25]. Pregabalin, an anticonvulsant drug used for neuropathic pain and later approved for treating generalized anxiety disorder (GAD), also showed similarities with multiple antidepressants (Fig. 1b). Pregabalin has shown efficacy in treating depressive symptoms associated with GAD as well [26].

Of the 1005 drugs we analyzed, 15 were immune-modulators and 66 were anti-inflammatory drugs. Of them, 7 immune-modulators and 14 anti-inflammatory drugs (7 COX inhibitors and 7 glucocorticoids) showed side effect similarities with antidepressants (Table 2). The enrichment is significant for the immune-modulators (Fisher exact test one-sided *P*-value = 0.015), but not for the anti-inflammatory drugs (*P*-value =0.75). Of the 7 COX inhibitors, only flurbiprofen had side effect similarities with more than one antidepressant. Four of the 7 glucocorticoids (hydrocortisone 17-butyrate, clocortolone, alclometasone and halcinonide) had side effect similarities with a single antidepressant, venlafaxine, forming a small cluster on the side effect similarity network (Fig. 1b). Two other glucocorticoids (cortisone and hydrocortisone) and one mentalocorticoid/glucocorticoid (fludrocortisone) had side effect similarities with lithium. Similar clusters were also formed by immune-modulator glatiramer acetate (GA) with 5 antidepressants, and between 4 immune-modulators and pramipexole. Drugs within these clusters share some rare side effects. For example, in the cluster formed by GA and neighboring 5 antidepressants, 10 rare side effects were shared by greater or equal to 3 drugs in addition to GA (Table 2). Some of these side effects are related to immune response (e.g. tenosynovitis which is an inflammation of the lining of the sheath that surrounds a tendon, and Rheumatoid arthritis) or mood disorders (e.g. depressive type psychosis), while others whose underlying biological processes may be more complicated (e.g. tongue discoloration).

**Table 2:** Drugs with side effect profiles similar to antidepressants

Drug	Drug type	Drug target	Indication	Neighboring antidepressant	Antidepressant’s mode of action	*P*-value*	Top shared rare side effects within cluster**
Glatiramer Acetate	Immune-modulator	HLA-DRB1	Multiple sclerosis	Fluoxetine	SSRI	0.001	Tenosynovitis (6); Skin hypertrophy (5); Tongue discolouration (5); Alcohol intolerance (4); Circumoral paresthesia (4); Dental caries (4); Depressive type psychosis (4); Rheumatoid arthritis (4); Salivary gland enlargement (4); Urethritis (4)
				Paroxetine	SSRI	0.006	
				Fluvoxamine	SSRI	0.001	
				Selegiline	MAO-B inhibitor	<0.001	
				Mirtazapine	5HT & α2-adrenergic receptor antagonist	<0.001	
Lenalidomide	Immune-modulator	TNF-α	Multiple myeloma; Myelodysplastic syndromes	Pramipexole	Non-ergoline dopamine agonist	0.019	Chronic obstructive pulmonary disease (3); Diverticulitis (3); Ear infection (3) Prostate cancer (3)
Ustekinumab	Immune-modulator	Interleukin 12/23	Plaque psoriasis; Psoriatic arthritis			0.002	
Pimecrolimus	Immune-modulator	Calcineurin	Atopic dermatitis			<0.001	
Siltuximab	Immune-modulator	Interleukin 6	Castleman’s disease			<0.001	
Rapamycin	Immune-modulator	Mammalian target of rapamycin (mTOR)	Rejection in organ transplant	Selegiline	MAO-B inhibitor	0.016	
Montelukast	Immune-modulator	Cysteinyl leukotriene receptor 1 (CYSLTR1)	Asthma; Seasonal allergies	Bupropion	Dopamine & norepinephrine reuptake inhibitor	0.011	
Naproxen	Anti-inflammatory; Analgesic	COX-1/2	Inflammations; Pain	Paroxetine	SSRI	0.006	Bleeding time prolonged (4); Retinal haemorrhage (4); Fever chills (4); Pyuria (4); Keratoconjunctivitis (3); WBC abnormal NOS (3); Tenosynovitis (3) ; Urethritis (3); Deficiency anaemia (3); Diarrhoea haemorrhagic (3); Electroencephalogram abnormal (3); Enteritis (3); Iron deficiency (3); Iron deficiency anaemia (3)
Ketoprofen				Fluoxetine	SSRI	0.003	
Flurbiprofen				Paroxetine	SSRI	<0.001	
				Fluoxetine	SSRI	<0.001	
Diclofenac				Nefazodone	5HT receptor antagonist	0.037	
Rofecoxib		COX-2		Duloxetine	SNRI	0.029	
Meloxicam				Clomipramine	Non-selective monoamine reuptake inhibitor (TCA)	0.031	
Valdecoxib				Sertraline	SSRI	0.015	
Fludrocortisone	Anti-inflammatory	Mineralocorticoid receptor/Glucocorticoid receptor	Addison’s disease/salt-losing adrenogenital syndrome	Lithium	Other	0.004	Benign intracranial hypertension (4); Exophthalmos (4); Papilloedema (4); Pseudotumor (4)
Cortisone		Glucocorticoid receptor	Inflammations; Pain			0.005	
Hydrocortisone		Glucocorticoid receptor	Severe allergic reactions; Inflammations			0.001	
Hydrocortisone 17-butyrate		Glucocorticoid receptor (NR3C1)	Skin irritations	Venlafaxine	SNRI	0.017	Miliaria (5); Skin atrophy (5); Skin striae (5)
Clocortolone						0.012	
Alclometasone						0.01	
Halcinonide		NR3C1 and SMO				0.026	

*P-value determined by random shuffling the side effect labels 1000 times and then compares the randomly generated cosine correlation coefficient with the original one

**The top shared rare side effects are the ones that are shared by more than half of the drugs sharing side effect similarity connections within the cluster. Numbers in the parentheses are the number of drugs within the cluster having that side effect. For clusters with a hub that has connections with all other drugs, the hub drug has all the top side effects listed

GA is a random polymer of 4 amino acids (TGAL copolymer) found in myelin basic protein. It binds to major histocompatibility complex II (MHC II) and competes with myelin antigens for their presentation to T cells. GA may increase brain-derived neurotrophic factor (BDNF) expression [27] and reduce P2X7 receptor level [28], both of which may play important roles in MDD [29, 30]. The interaction map of GA from Ingenuity shows that proteins and complexes known to interact with GA directly or indirectly are highly enriched for genes involved in nervous system development and function (*p*-value of right-tailed Fisher Exact test =7.3 E-23).

Four of the 7 immune-modulators had side effect similarities with pramipexole, a dopamine receptor agonist with antidepressant activity approved for treating Parkinson’s disease. . These 4 drugs are immune-modulators with different targets: lenalidomide targeting TNF-α, ustekinumab targeting interleukin 12 and 23, siltuximab targeting interleukin 6 and pimecrolimus inhibiting calcineurin. It has shown antidepressant activity in multiple controlled clinical trials as well as open-label studies [23]. Another immune modulator, montelukast, also showed side effect similarity with antidepressant targeting the dopamine pathway. Montelukast is a drug used for treating asthma and seasonal allergies. It showed side effect similarities with antidepressant bupropion, which is a dopamine and norepinephrine reuptake inhibitor (Fig. 1b). Montelukast is a leukotriene receptor antagonist, and has been shown to be associated with possible increase in suicidal behavior and depression [31].

Rapamycin showed side effect similarity with antidepressant selegiline (Fig. 1). Rapamycin is used as an immunosuppressant to prevent rejection after organ transplant. It inhibits mTOR (mechanistic Target of Rapamycin), which is involved in translation control and long-term synaptic plasticity [32]. Recent studies showed that chronic partial inhibition of mTOR by rapamycin reduce anxiety and depressive-like behavior in mice, possibly by stimulating major monoamine pathways [33], whereas intracerebroventricular infusion of rapamycin could inhibit the rapid antidepressant activity of ketamine [34].

### Connectivity map analysis

Similarities in gene expression profiles of human cells treated with bioactive small molecules has also been explored as a method for identifying drugs sharing common mechanism of action [19]. To further explore the relationship between drugs included in our study, we queried the Connectivity Map (CMAP) database for our 7 immune-modulators and 14 anti-inflammatory drugs. Of the 7 immune-modulators, only rapamycin (Sirolimus) had expression profiles in CMAP. There are 44 “instances” in CMAP recording the changes in different human cell lines induced by rapamycin. Each instance contains a treated and control cell pair. To reduce the influence of experimental batch and cell lines, we selected instances from the same batch and cell lines and generated the expression signatures by selecting the genes that showed at least 1.5 fold of change between cells treated with rapamycin and control cells. These generated 12 rapamycin expression profiles from 36 CMAP instances. Each expression profile contains a list of up/down regulated genes in the rapamycin treated cell lines. We then queried the database for drugs with similar expression profiles to these 12 rapamycin profiles using the pattern-matching tool provided by CMAP. Two profiles from PC3 cells and 5 profiles from MCF7 cells treated with rapamycin were similar to profiles of 2 types of antidepressants (permutation *p*-value of enrichment <0.05): non-selective monoamine reuptake inhibitors such as clomipramine, amoxapine, protriptyline, maprotiline, desipramine, nortriptyline, trimipramine, and amitriptyline; and selective serotonin reuptake inhibitors such as zimelidine and fluoxetine (Table 3). However, 3 profiles from HL60 cells and 2 profiles from MCF7 cells treated with rapamycin did not show similarities with antidepressants. The same query was conducted for the 14 anti-inflammatory drugs. All except 2 (meloxicam and clocortolone) had instances in CMAP database. Of them, alclometasone showed expression similarities with antidepressants fluvoxamine and citalopram (Table 4).

**Table 3:** CMAP expression signature similarities between human cells treated with rapamycin and antidepressants

CMAP Instance^a^	Cell	Number of up-regulated probes^b^	Number of down-regulated probes^b^	Antidepressant with similar expression signature	Antidepressant Type	*P*-value of expression signature similarity	Enrichment score^c^
4431; 4445; 4466	PC3	108	112	clomipramine	non-selective monoamine reuptake inhibitors	0.04844	0.627
1207; 1221; 1242	PC3	84	82	amoxapine	non-selective monoamine reuptake inhibitors	0.01139	0.664
				protriptyline		0.01156	0.724
				maprotiline		0.01993	0.688
987; 1001; 1022	MCF7	27	56	desipramine	non-selective monoamine reuptake inhibitors	0.01156	0.724
				zimeldine	selective serotonin reuptake inhibitors	0.00537	0.704
				fluoxetine		0.03	0.661
1045; 1059; 1080	MCF7	4	182	clomipramine	non-selective monoamine reuptake inhibitors	0.01017	0.732
				protriptyline		0.00346	0.795
				nortriptyline		0.00109	0.837
5567; 5581; 5602	MCF7	125	67	amoxapine	non-selective monoamine reuptake inhibitors	0.03467	0.593
				trimipramine		0.04345	0.635
				nortriptyline		0.0219	0.682
6967; 6981; 7001	MCF7	44	51	clomipramine	non-selective monoamine reuptake inhibitors	0.0157	0.705
				protriptyline		0.02538	0.672
				nortriptyline		0.0192	0.69
6927; 6940; 6958	MCF7	25	60	amitriptyline	non-selective monoamine reuptake inhibitors	0.01422	0.596

^a^The ID of the instances from CMAP database used to generate the query expression signature for rapamycin

^b^Number of up and down regulated probes of the querying expression signature determined after 1.5 fold change in expression level between drug-treated cells and control cells

^c^A measure of the enrichment of the instances for antidepressant in the list of all instances ordered by expression profile similarity score

**Table 4:** CMAP expression signature similarities between human cells treated with alclometasone and antidepressants

CMAP instance	Drug	Cell	Number of up-regulated probes	Number of down-regulated probes	Antidepressant with similar expression signature	Antidepressant type	*P*-value of expression signature similarity	Enrichment score
6229; 6094	Alclometasone	MCF7	58	35	fluvoxamine	selective serotonin reuptake inhibitors	0.0174	0.697
					citalopram		0.0247	0.674

## Discussion

In this study, we’ve explored the space of drug side effect profile similarities between immune-modulators/anti-inflammatory medications and antidepressants. Side effect profiles contain complex phenotypic information related to the underlying physiological pathways affected by medications. Similarities in side effect profiles indicate that drugs share common targets or off-targets, or perturb common downstream pathways. Thus this could provide us new insights as to how the immune system may influence mood and *vice versa*. The immune-modulators and anti-inflammatory drugs sharing side effects with antidepressants in our study fall into two different categories: one targeting pathways such as glucocorticoid signaling, which is known to affect both immune and nervous systems; the other targeting mainly pro-inflammatory mediators such as cytokines, TNF-α and calcineurin, which may then influence the nervous system through a bidirectional communication route between the immune and the nervous system.

Four glucocorticoids showed side effect similarity with antidepressant venlafaxine while another three showed similarity with lithium in our study. One of them, alclometasone induced expression profiles in human cells similar to that induced by antidepressants fluvoxamine and citalopram, suggesting that glucocorticoids and antidepressants may induce common molecular pathways. Glucocorticoids exert their anti-inflammatory effect by binding to glucocorticoid receptor (GR), which then suppress the transcription of pro-inflammatory cytokines such as IL-6, IL-12, COX-2 and TNFα [35]. GR has also been proposed to play pivotal role in depression and antidepressant treatment [36]. Under normal physiological conditions, glucocorticoid activated GR mediates a negative feedback loop to maintain low glucocorticoid levels in the brain. However, this negative feedback loop is impaired in some depressed patients, resulting in chronic high levels of glucocorticoids and constant hyperactivity of the hypothalamus-pituitary-adrenal (HPA) axis [37]. Studies in rodent models have shown that chronic glucocorticoid treatment induces depression and anxiety-like behavior, and reduction of adult hippocampal neurogenesis [38]. Treatment with antidepressants could normalize HPA axis hyperactivity, possibly by restoring GR function [36]. Interestingly, glucocorticoids such as prednisolone, dexamethasone and cortisol have been reported to have antidepressant-like effects in MDD patients, possibly by restoring the negative feedback loop on the HPA axis [39–41]. Taken together, these suggest that the glucocorticoid receptor is involved in both immune regulation and depression, and may be a viable target for treating depressed patients with HPA axis hyperactivity.

Another example is glatiramer acetate (GA), which has a dual anti-inflammatory and neuroprotective role in treating multiple sclerosis (MS) [42]. GA is a synthetic polypeptide mixture containing amino acids glutamic acid, lysine, alanine and tyrosine in a molar ratio of 1.4 : 3.4: 4.2 : 1.0 assembled in a random order into polypeptide chains with 40 ~ 100 amino acids [43]. Peripheral GA treatment (either by subcutaneous injections or by oral administration) induces GA-specific T cells in the peripheral immune system, which can cross the blood brain barrier to accumulate in the CNS. These T cells are type 2 T helper (Th2) cells that secrete anti-inflammatory cytokines such as IL-4, IL-10 and TGF-β, thus blocking the immune response in the CNS. In addition, Th2 cells also produce neurotrophic factors such as BDNF, which helps to rescue degenerating neurons and promote neural regeneration [44]. Furthermore, GA-specific T cells also induced a “bystander effect” on CNS resident cells to produce anti-inflammatory cytokines and neurotrophic factors [43]. It is interesting to observe that among the immune-modulators we studied, GA is the only one that shows side effect profile similarity with multiple antidepressants. However, it is unknown to us whether these similarities were the result of anti-inflammatory effect of GA or its neuroprotective effect, or both. Because antidepressant treatment, like GA, can also restore the decreased BDNF function in MDD patients up to the normal level [45]. According to this evidence, the dual effect of GA treatment may also benefit patients with depressive symptoms and is worth further study.

Rapamycin, also known as sirolimus, may also have dual functions regulating both the immune and nervous system. Rapamycin forms a complex with FK-binding protein 12 (FKBP12) and then inhibits the mTOR pathway by binding directly to mTOR complex 1. The mTOR pathway is involved in both immune response regulation [46] and synaptic plasticity [47] by acting as a bridge between extracellular signals and translation machinery. Inhibition of mTOR by rapamycin can block the response to IL-2, thus suppressing immune system. Interestingly, blocking the mTOR pathway by intracerebroventricular infusion of rapamycin could inhibit the rapid antidepressant activity of ketamine [34], whereas chronic and partial inhibition of mTOR by rapamycin could reduce anxiety and depressive-like behavior in mice throughout their lifespan, possibly by stimulating the production of major monoamines (norepinephrine, dopamine and 5-hydroxytrptamine) [33]. Our study showed that at both the clinical level and cellular level, rapamycin shared phenotypic similarities with antidepressants targeting monoamines: monoamine oxidase B (MAO-B) inhibitor, selegiline, which prevents the breakdown of monoamines; as well as monoamine reuptake inhibitors, which helps increase the extracellular concentrations of the monoamine neurotransmitters. Besides the regulation of monoamine levels, another possible link between MAO-B activity and rapamycin is mitochondria turnover. One study has found that age or neurodegenerative disorder related brain MAO-B level elevation could contribute to the accumulation of damaged mitochondrial and neurodegeneration. Treatment by rapamycin could counteract this process by enhancing autophagic removal of damaged mitochondria [48]. Based on current knowledge, the immune suppressant activity and antidepressant-like activity of rapamycin may both depend on its ability to inhibit the mTOR pathway. Further analyses are needed to reveal whether the antidepressant-like activity of rapamycin is dependent on its immune suppressant activity.

In our study, most of the immune-modulators sharing side effect profile similarities with antidepressants do not seem to have dual functions like GA and rapamycin. They suppress the immune response by targeting pro-inflammatory mediators. These immune-modulators had side effect profiles similar to antidepressants targeting the dopamine pathway. Four of them had side effect similarities with Pramipexole, a dopamine receptor agonist with antidepressant-like activity. Another drug, montelukast, a leukotriene receptor antagonist, showed side effect similarities with antidepressant bupropion, which is a norepinephrine-dopamine reuptake inhibitor. Dopamine has been proposed as one of the key neurotransmitters bridging the nervous and immune systems, since dopamine and its receptor are expressed not only by the central and peripheral nervous cells, but also by different immune cells [49]. Communication between CNS and the peripheral immune system may be carried out by dopamine activated T cells crossing the blood brain barrier [50]. A recent study showed that electroacupuncture at the sciatic nerve of mice could induce dopamine production in the adrenal medulla, which then leads to the inhibition of cytokine production by dopamine, confirming the dopaminergic regulation of immune response [51]. Interestingly, studies have shown that changes in the expression of dopamine receptors and their signaling pathways, especially in T cells, are associated with altered immune functions in schizophrenia and Parkinson’s disease [52, 53]. The similarities in the side effect profiles as observed by us may be a reflection of the communication between immune system and nervous system through dopamine. Even though deficiencies in dopamine, serotonin and noradrenaline are thought to underpin MDD pathophysiology, treating MDD with drugs targeting dopamine pathway is under-recognized currently [54]. Considering the role that dopamine plays in both mood and immune response, it may provide viable treatment options for MDD patients who are resistant to treatments with SSRI, and who have elevated immune activation, perhaps indicated by CRP levels.

Like dopamine, serotonin has also been shown to be able to act as signaling agents between immune cells, helping to enhance T cell activation [55, 56]. There is also evidence supporting that CNS serotonin neurons are direct or indirect targets for the communication from the immune system to the brain via both humoral and neuronal mechanisms [57]. The pro-inflammatory cytokine IL-1β could increase extracellular levels of serotonin in the brain as well as serotonin transporter (SERT) activity, which may reflect a coordinated serotonergic response to immune activation, and disruption of this homeostatic mechanism may be a risk for diseases such as depression or autism [57]. Seven COX inhibitors in our study showed side effect similarities with antidepressants, which targets the serotonin signaling (Table 2). Our results seem to indicate that COX inhibitors targeting the synthesis of prostanoids and immune-modulators targeting other pro-inflammatory mediators may interact with nervous systems through different communication routes. Whether this is related to differences between the serotonergic and dopaminergic systems needs further explorations.

Interestingly, studies have shown that antidepressants also have anti-inflammatory effects, possibly through increasing production of IL-10 [58] and suppressing the expression of TNF-α and IL-6 [59]. This is consistent with our findings that antidepressants and immune-modulators may affect similar molecular mechanisms and thus share common physiological effects, and further supports the idea that serotonin and other monoamines targeted by antidepressants may play an important role in the communication between the immune and nervous system. Several immune-modulators and anti-inflammation medications have also shown antidepressant-like effects [14]. For example, IL-12/23 inhibitor ustekinumab improves symptoms of depression and anxiety in patients with psoriasis [10]. COX 2 inhibitors such as celecoxib have been reported to benefit MDD patients when used together with other antidepressants but not when using alone [11, 13]. TNF-α inhibitor infliximab could reduce depressive symptoms in treatment-resistant MDD patients having relatively more severe inflammation symptoms [9]. In our analysis, ustekinumab showed side effect similarities with antidepressants, but celecoxib and infliximab did not. This could mean that antidepressants in our study share limited common molecular mechanisms with celecoxib and infliximab. But it could also due to incomplete side effect profiles captured by the drug labels of these drugs. Even though we analyzed the side effects of over 1000 drugs, it is by no means a comprehensive survey of all marketed drugs. Additional similarities between antidepressants and immune related medications may be revealed with a bigger drug-side effect database. A more comprehensive survey of the side effects reported from more patients such as those stored in the FDA adverse reporting system may help resolve this question in the future. Another approach is to extend the side effect profiles using in silico side effect prediction methods based on correlation between side effect and drug structure [60] or based on predicted interactions between drugs and key metabolic enzymes such as cytochrome P450 [61].The value of repurposing drugs has to be explored in the context of the side-effect risk associated with the repurposed compound; for example biologics are often associated with higher risk of infections, such as pneumonia [62]; therefore the value proposition for the patient would be based on having failed to respond to safer compounds, and ideally the ability to enrich for patients likely to respond. Identifying that cohort is beyond the scope or capability of this study, but an essential next step. Nevertheless, results from our side-effect profile similarities suggest that the antidepressant potential of several novel candidates such as Siltuximab and Pimecrolimus may worth further exploration, as may the re-evaluation of dopaminergic antidepressants among MDD patients with high levels of inflammation.

## Conclusions

Our analysis of drug-similarity based on side effect profiles indicates that some antidepressants and some immune-related drugs may affect common molecular pathways. This may result from the immune-modulators affecting mood directly through the drugs intended target, indirectly through downstream effects on serotonin and/or dopamine pathways, or through novel mechanisms not yet understood. Our findings support the idea that certain medications aimed at the immune system may be helpful in relieving depressive symptoms, and suggest that it may be of value to test immune-modulators, such as rapamycin and alclometasone, for antidepressant-like activity in future proof-of-concept studies. Our results also support the hypothesis that both dopamine and serotonin may play critical roles in the communications between immune and CNS systems. For MDD patients who fail to respond to treatment with antidepressants aiming at the serotonergic pathway, compounds targeting the dopaminergic pathway may provide new choices for the subset of patients with an inflammatory diathesis. Side effect profile based drug-drug similarity is particularly informative, since side effect profile is derived from drug intervention in human. However, this metric provides only one view of the crosstalk between depression and immune response. Therefore, to further clarify the mechanistic similarities between antidepressants and drugs targeting the immune response system, analysis of additional drug similarity metrics base on drug structure, treatment elicited gene expression signatures, pathway enrichment, and protein-protein interaction networks are needed. These data may inform novel mechanisms and identify new targets for antidepressant effects, and may aid in the prioritization process for designing more direct proof-of-concept studies.

## Abbreviation

BDNF: Brain-Derived Neurotrophic Factor
COX: Cyclooxygenase
CRP: C-reactive proteins
GA: Glatiramer Acetate
GAD: Generalized Anxiety Disorder
GR: Glucocorticoid Receptor
HPA: Hypothalamus-Pituitary-Adrenal
IL-1: Interleukin 1
IL-6: Interleukin 6
MAO-B: MonoAmine Oxidase B
MDD: Major Depressive Disorder
MHC II: Major Histocompatibility Complex II
MS: Multiple Sclerosis
mTOR: mechanistic Target of Rapamycin
NSAIDs: NonSteroidal Anti-Inflammatory Drugs
SERT: Serotonin Transporter
SNRI: Serotonin-Noradrenaline Reuptake Inhibitors
SSRI: Selective Serotonin Re-uptake Inhibitors
TCA: TriCyclic Antidepressants
TeCA: TetraCyclic Antidepressants
TNF-α: Tumor Necrosis Factor alpha
